# 

**Published:** 1906-09-01

**Authors:** 


CONTENTS.

London as a Medical Centre - - - - - - - 375
London as a Surgical Teaching Centre - - - - 376
Qualification and Registration - - - - - - - 379
Notes on Medical Schools and Colleges - - - - 380
Handbooks for Medical Students - - - - - - 385
Literary Workers - - - - - - - - - - 386
Opportunities for Recent Graduates - - - - 387
Schools for Recent Graduates - - - - - - 388
Post-Graduate Opportunities in Special Hospitals 391
Occupation for the Recent Graduate - - - - 393
The Study of Tropical Medicine - - - - - 395

*NURSING SECTION.*

Notes on News from the Nursing World—Our Clothing Distribution—In Defence of Outdoor Uniform—Guardians and Grievances—The Future Home of the Royal National Pension Fund—More Trouble at Waihi Hospital—Full Value for Money—Another Phase of the Holiday Question—One Night Nurse for Two Blocks—Cricket and District Nursing—Nurses' Social Union—St. Katherine's Hospital—Short Items - - - - - - - - - - - 309–311
The Nursing Outlook - - - - - - - - 312
The Care and Nursing of the Insane - - - - - 313
The Nurses' Clinic - - - - - - - - - 314
Incidents in a Nurse's Life - - - - - - - 315
Visit to a Famous Irish Hospital - - - - - 316
Should there be Training Schools for Matrons? - 317
"In Coster Clubland" - - - - - - - - 318
The Final Cookery Lesson - - - - - - - 318
Everybody's Opinion - - - - - - - - 319
Appointments - - - - - - - - - - 320
The Nurses' Bookshelf - - - - - - - - 320
A Book and Its Story - - - - - - - - 321
Notes and Queries - - - - - - - - - 322
For Reading to the Sick - - - - - - - 322

